# Wall Thickness‐Guided vs. Voltage‐Guided Pulmonary Vein Isolation for Atrial Fibrillation

**DOI:** 10.1002/joa3.70215

**Published:** 2025-10-27

**Authors:** Moyuru Hirata, Ryuta Watanabe, Koichi Nagashima, Yuji Wakamatsu, Naoto Otsuka, Shu Hirata, Yuji Saito, Masanaru Sawada, Shiro Nakahara, Yasuo Okumura

**Affiliations:** ^1^ Division of Cardiology, Department of Medicine Nihon University School of Medicine Tokyo Japan; ^2^ Department of Cardiology Dokkyo Medical University Saitama Medical Center Koshigaya Japan

**Keywords:** atrial fibrillation, very high‐power short‐duration ablation, voltages, wall thickness

## Abstract

**Background:**

The efficacy and safety of tailored pulmonary vein isolation (PVI) guided by either left atrial wall thickness (LAWT) or bipolar voltage remain unclear.

**Objective:**

The aim of this prospective study was to evaluate the efficacy and safety of each ablation strategy.

**Methods:**

We conducted a prospective analysis of 97 patients with non‐valvular atrial fibrillation (AF) who underwent an initial RF catheter ablation procedure known as an extensive encircling PVI. Fifty patients underwent PVI using a wall thickness (WT)‐guided approach using ADAS 3D software and 47 patients using a voltage‐guided approach. In each strategy, high‐power short‐duration (HPSD) ablation was applied to regions with increased LAWT or elevated bipolar voltage, respectively, while very high‐power short‐duration (vHPSD) ablation was delivered to the remaining regions.

**Results:**

The first‐pass PVI rate tended to be higher in the WT‐guided group compared to the Voltage‐guided group (43 [86%] vs. 34 [72%], *p* = 0.09), and the incidence of acute PV reconnection (APVR) tended to be lower (5 [10%] vs. 11 [23%], *p* = 0.07). The proportion of patients with PV gaps (defined as the combined occurrence of first‐pass failure and/or APVR) was significantly lower in the WT‐guided group (10 [20%] vs. 18 [38%], *p* = 0.04). The multivariable‐adjusted analysis demonstrated that WT‐guided ablation was significantly more effective than Voltage‐guided ablation in preventing PV gaps. Both ablation strategies were performed without any procedural complications.

**Conclusions:**

WT‐guided ablation was associated with a significantly lower incidence of PV gaps than a conventional bipolar voltage‐guided strategy.

## Graphical abstract

Wall‐thickness guided pulmonary vein isolation using integrated pre‐procedural CT imaging and ADAS 3D software was associated with a significantly lower incidence of PV gaps than a conventional bipolar voltage‐guided strategy despite similar 1‐year clinical outcomes. These findings support individualized, left atrial wall thickness–based strategies in the high‐power RF ablation era.

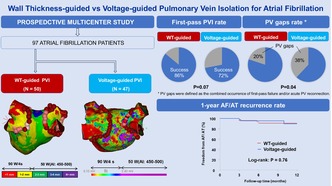

## 1 Introduction

Recent evidence has substantiated the safety and efficacy of pulmonary vein isolation (PVI) for atrial fibrillation (AF) using high‐power, short‐duration (HPSD) radiofrequency ablation strategies, typically employing power settings of 40–50 W for 10–15 s [1]. A novel modality, very high‐power short‐duration (vHPSD) ablation using the QDOT MICRO catheter, has been introduced with the goal of further reducing procedural time while preserving safety and therapeutic efficacy [2]. Clinical investigations have demonstrated that vHPSD ablation with this catheter significantly shortens total ablation time and achieves comparable safety and efficacy outcomes to those of conventional HPSD ablation [3, 4, 5]. Nonetheless, concerns have been raised regarding a modestly lower first‐pass PVI rate and an increased incidence of acute pulmonary vein (PV) reconnection associated with the vHPSD approach compared to HPSD [3, 4]. In a previous retrospective analysis, we found that the sites where gaps occurred during PVI using vHPSD exhibited higher bipolar voltages (≥ 2.4 mV) and thicker left atrial (LA) wall regions (≥ 2.3 mm). Multivariate analysis identified these parameters as the strongest independent predictors of PV gaps [6]. Traditionally, catheter ablation has been performed using 3D mapping systems that represent the three‐dimensional structure of the LA and pulmonary veins (PVs) based on pre‐procedural computed tomography (CT) images. However, conventional 3D mapping systems do not incorporate atrial wall thickness information. This study aimed to establish an individualized and efficient ablation strategy by integrating left atrial wall thickness (LAWT) information derived from pre‐procedural CT imaging with ADAS 3D software (ADAS 3D, Galgo Medical, Barcelona, Spain) into the 3D electroanatomical mapping system, or alternatively, by utilizing real‐time bipolar voltage data.

## 2 Methods

### 2.1 Study Design

We conducted a prospective, non‐randomized observational study involving 100 consecutive patients with non‐valvular AF who underwent an initial radiofrequency (RF) catheter ablation procedure—specifically, extensive encircling PVI (EEPVI)—at Nihon University Itabashi Hospital and Dokkyo Medical University Saitama Medical Center between December 2023 and March 2025. All patients were eligible for inclusion if they were aged ≥ 20 years, had symptomatic paroxysmal or persistent non‐valvular AF, and were scheduled to undergo a first‐time EEPVI procedure. Exclusion criteria included a history of prior LA ablation, valvular AF, LA thrombus, contraindications to anticoagulation therapy, or inability to provide informed consent.

**FIGURE 1:**
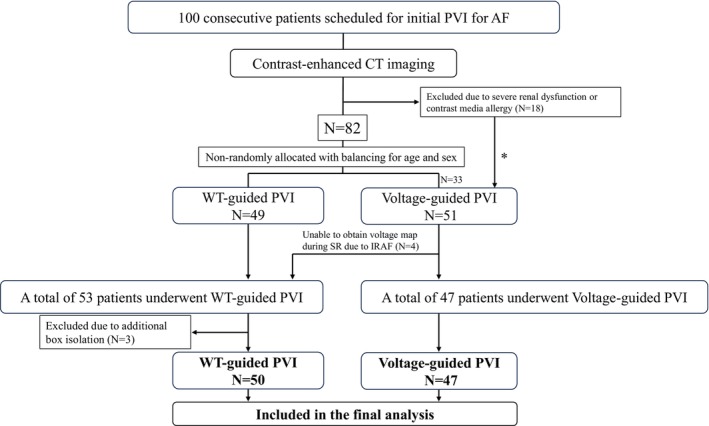
Study flow diagram of patients undergoing initial PVI for atrial fibrillation. Details are shown in the text. *The initial 18 patients without CT were added to the voltage‐guided group (total *n* = 51). AF, atrial fibrillation; CT, computed tomography; IRAF, immediate recurrence of atrial fibrillation; PVI, pulmonary vein isolation; SR, sinus rhythm; WT, wall thickness.

Preprocedural contrast‐enhanced cardiac CT was planned 1–7 days before the ablation procedure. However, 18 patients were unable to undergo CT due to severe chronic kidney disease (CKD) or known contrast media allergy. The remaining 82 patients underwent CT and were non‐randomly allocated to the wall thickness‐guided PVI group (WT‐guided PVI, *n* = 49) or voltage‐guided PVI group (*n* = 33), while balancing age and sex between groups. The 18 patients without contrast‐enhanced CT were assigned to the Voltage‐guided group, resulting in a total of 51 patients in that group. During the procedure, four patients in the Voltage‐guided group experienced immediate recurrence of AF (IRAF), which precluded voltage mapping during sinus rhythm (SR). These patients were reassigned to the WT‐guided PVI group. Thus, 53 patients underwent WT‐guided PVI and 47 underwent voltage‐guided PVI. Among those in the WT‐guided group, three patients underwent additional box isolation and were excluded from the final analysis. Ultimately, 50 patients in the WT‐guided group and 47 in the voltage‐guided group were included in the final analysis. A schematic representation of the study flow is shown in Figure 1.

All patients received adequate oral anticoagulation therapy for at least 1 month prior to the ablation procedure. The study protocol was approved by the Institutional Review Board of Nihon University Itabashi Hospital (RK‐230314‐14).

### 2.2 Three‐Dimensional Computed Tomography (CT) Image From ADAS Software

Multi‐detector helical CT with a 320‐row detector and dynamic volume CT scanner (Aquilion ONE; Toshiba Medical Systems, Tokyo, Japan) was performed at a slice thickness of 0.5 mm, gantry rotation time of 350 ms, tube voltage of 120 kV, and tube current of 300–580 mA for optimum detection of delicate structures (resolution of approximately 0.3 mm). Landiolol was administered to maintain the patient's heart rate at < 65 bpm, and nonionic iodinated contrast (Iomeron, Eisai Co, Tokyo, Japan) was injected at 0.07 mL/kg/s for 9 s. The timing of the image acquisition was determined by bolus tracking software; imaging was initiated when the contrast reached LA. During the end‐expiratory phase, the volume acquisition was gated to 65%–75% of the R‐R interval on the lead II electrocardiogram during SR or an AF rhythm.

**FIGURE 2:**
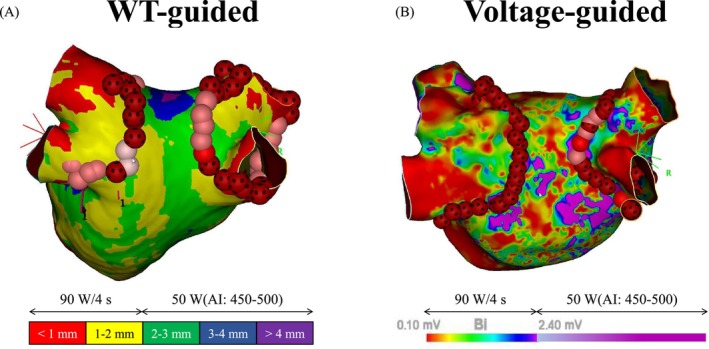
Representative protocols for Wall thickness–guided (A) and Voltage‐guided ablation strategies (B). WT, Wall thickness.

The acquired CT data were processed using the ADAS 3D software to reconstruct a 3D image (ADAS 3D image) incorporating LAWT information (Figure 2). In the ADAS 3D image, LAWT is color‐coded as follows: red for < 1 mm, yellow for 1–2 mm, green for 2–3 mm, blue for 3–4 mm, and purple for ≥ 4 mm. The reconstructed ADAS 3D image was subsequently integrated into the CARTO system, a 3D electroanatomical mapping system in 82 patients.

### 2.3 Mapping and Ablation Procedure

An electrophysiologic study was performed with patients under conscious sedation achieved with dexmedetomidine and fentanyl, with additional propofol administered if needed. After vascular access was obtained, a single transseptal puncture was performed, and intravenous heparin was administered to maintain an activated clotting time of > 300 s. After two long sheaths (VIZIGO steerable sheath; Biosense Webster, Diamond Bar, CA, USA and SL0 sheath; Abbott Medical Japan G.K., Tokyo, Japan) were inserted into the LA via a transseptal puncture, the 3‐dimensional (3D) geometry of the LA and 4 PVs was reconstructed using a CARTO3 mapping system (Biosense Webster, Diamond Bar, CA, USA). The geometry and electroanatomical mapping of the LA and PVs were created in all patients. During mapping, a high‐pass filter of 30 Hz and a low‐pass filter of 500 Hz were applied. A multi‐electrode, multi‐spline catheter with 2 mm interelectrode spacing (OCTARAY NAV, Biosense Webster) was used.

In this study, EEPVI was performed using a combination of HPSD and vHPSD settings, guided by LAWT or bipolar voltage. The decision to perform the procedure using either a WT‐guided or voltage‐guided approach was at the discretion of the operator. Ablation was conducted using an irrigated catheter (QDOT MICROTM catheter, Biosense Webster, Diamond Bar, CA) with a contact force (CF) of 5–20 g and an inter‐lesion distance (ILD) of < 6 mm. Temperature‐controlled ablation was performed using the QDOT MICRO catheter with an RF generator (nGEN RF Generator, Biosense Webster).

### 2.4 Ablation Protocol

#### 2.4.1 Wall Thickness‐Guided Ablation

In the WT‐guided group, LA geometry and bipolar voltage mapping were reconstructed irrespective of cardiac rhythm, including SR, AF, and AT. EEPVI was performed using HPSD and vHPSD settings according to the LAWT visualized on the ADAS 3D image integrated into the 3D mapping system. Specifically, regions with a LAWT ≥ 2 mm (represented in green, blue, or purple on the ADAS 3D image) were ablated using the HPSD setting, while regions with a LAWT < 2 mm (represented in red or yellow) were targeted with the vHPSD setting (Figure 2A). The HPSD setting involved delivering RF energy at 50 W, targeting an ablation index (AI) of 450–550. The vHPSD set applied RF energy at 90 W for 4 s. The acquired bipolar voltage data were used exclusively for retrospective analysis of PV gaps and were blinded to the operator during the procedure.

#### 2.4.2 Voltage‐Guided Ablation

In the Voltage‐guided group, a PV‐LA high‐density 3D bipolar voltage map during SR was created before ablation. In cases where the patient was in AF, synchronized low‐energy electrical cardioversion (10–20 J) was performed to restore SR, followed by creating the high‐density 3D bipolar voltage map. The bipolar voltage map was configured such that areas with bipolar voltage ≥ 2.4 mV were displayed in purple. HPSD was applied to regions with a bipolar voltage ≥ 2.4 mV (represented in purple on the bipolar voltage map), and vHPSD was applied to regions with a voltage < 2.4 mV (represented by colors other than purple) (Figure 2B). The HPSD setting involved delivering RF energy at 50 W, targeting an AI of 450–550. The vHPSD set applied RF energy at 90 W for 4 s. The acquired ADAS 3D CT data were used exclusively for retrospective analysis of PV gaps and were blinded to the operator during the procedure.

In both groups, the procedures were performed by six experienced operators in AF ablation. Among them, three operators had performed more than 500 procedures and three operators had performed more than 100 procedures.

### 2.5 Verification of Pulmonary Vein Isolation

Completion of PVI was confirmed by demonstration of the bidirectional block. Entrance block was defined by the disappearance of PV potentials, while exit block was confirmed by the absence of conduction to the LA during bipolar pacing from the PV ostium using an OCTARAY catheter. Successful isolation of the PVs with a single circumferential ablation set was defined as first‐pass PVI. If first‐pass PVI was not achieved, residual conduction gaps were identified using a mapping catheter and ablated using the HPSD setting to complete the PVI. A 30‐min observation period was performed after PVI. In cases where spontaneous PV reconnection was observed, additional ablation was performed to re‐establish isolation. Subsequently, rapid intravenous administration of adenosine triphosphate (ATP) was conducted to assess for dormant conduction. Further ablation was applied to achieve complete PVI if dormant conduction was detected. The presence of either spontaneous reconnection or dormant conduction was defined as acute pulmonary vein reconnection (APVR). The first‐pass PVI failure and/or APVR was defined as the PV gaps.

### 2.6 Follow‐Up

Post‐ablation antiarrhythmic drug was determined at the operators' discretion based on individual patient characteristics. The patients at Nihon University Itabashi Hospital and Dokkyo Medical University Saitama Medical Center underwent routine follow‐up at 3 weeks, 3, 6, 12, and subsequently every 6 or 12 months post‐ablation or whenever they experienced any arrhythmia‐related symptoms. During each visit, 12‐lead electrocardiograms were recorded, and 24‐h Holter monitoring was performed at 3–6 months, 12, and every 12 months post‐ablation.

Recurrence was defined as any documented AF or atrial tachycardia (AT) lasting more than 30 s during the follow‐up period.

### 2.7 Study Outcomes

The primary effectiveness outcome of this study was the success rate of first‐pass PVI. The PV gaps (defined as the combined occurrence of first‐pass failure and/or APVR) were also compared between the WT‐guided and Voltage‐guided groups. The primary safety outcome was procedure‐related complications within 2 days of the ablation. The secondary outcome was the 1‐year AF/AT freedom rate after a 3‐month blanking period. The distribution of PV gaps was evaluated by dividing the region into the following 16 PV segments: the anterior, posterior, and superior aspects of the right and left superior PVs (RSPV and LSPV, respectively), anterior and posterior carinas of the right and left PVs, and anterior, posterior, and inferior aspects of the right and left inferior PVs (RIPV and LIPV, respectively).

### 2.8 Statistical Analysis

Continuous variables were expressed as mean ± standard deviation or as median with interquartile ranges (25th and 75th percentiles), as appropriate. Categorical variables were presented as counts and percentages. Differences in continuous variables were analyzed using either the Student's *t*‐test or the Mann–Whitney *U* test, depending on data distribution. Differences in dichotomous variables were assessed using the chi‐squared test; if the expected cell count was < 5, Fisher's exact test was employed. The Kaplan–Meier method generated the AF/AT recurrence‐free survival curve and compared it using a log‐rank test between the WT‐guided and Voltage‐guided groups. Univariate and multivariate logistic regression analysis for the PV gap was conducted in ablation strategies (WT‐guided or Voltage‐guided), RF output setting (90 W or 50 W), carina or non‐carina, and LAWT > 2 mm respectively. In all cases, ablation tags were retrospectively assigned to predefined PV segments for subsequent analysis. Additionally, in the 79 patients who underwent contrast‐enhanced CT (3 patients excluded due to additional box isolation from 81 patients who underwent contrast‐enhanced CT image), three‐dimensional CT reconstructions incorporating LAWT data were generated using the ADAS 3D software, irrespective of group assignment. The LAWT range for each segment was retrospectively measured. All statistical analyses were performed using JMP Pro 17 software (SAS Institute Inc., Cary, NC, USA).

## 3 Results

### 3.1 Patient Characteristics and Echocardiographic Findings

The clinical characteristics of the study population are summarized in Table 1. Of the 97 patients enrolled, 61 (63%) had paroxysmal atrial fibrillation (PAF), and 36 (37%) had persistent atrial fibrillation (PerAF). The median CHA2DS2‐VASc score was 2 (interquartile range: 1–3). The mean LA diameter (LAd) was 39 ± 6 mm, and the mean left ventricular ejection fraction (LVEF) was 64% ± 11%. The WT‐guided group had a greater prevalence of comorbidities such as heart failure compared to the Voltage‐guided group (14 [28%] vs. 5 [10%], *p* = 0.04). The echocardiographic evaluation revealed that the WT‐guided group had a significantly lower LVEF (61% ± 13% vs. 66% ± 8%, *p* = 0.01) and a significantly larger LAd (40 ± 6 mm vs. 38 ± 6 mm, *p* = 0.03) than the Voltage‐guided group.

**TABLE 1:** Patient characteristics.

	Total (*n* = 97)	WT‐guided (*n* = 50)	Voltage‐guided (*n* = 47)	*p*
Clinical characteristics				
Age (years)	66 ± 11	65 ± 10	66 ± 11	0.79
Male sex	63 (64%)	30 (60%)	33 (70%)	0.29
BMI (kg/m^2^)	23 ± 4	24 ± 5	24 ± 4	0.98
Paroxysmal AF	61 (63%)	21 (42%)	15 (31%)	0.30
AF duration (months)	19 ± 37	25 ± 47	14 ± 22	0.17
Hypertension	51 (53%)	27 (54%)	24 (51%)	0.77
Hyperlipidemia	13 (13%)	7 (14%)	6 (12%)	0.85
Diabetes mellitus	15 (15%)	10 (20%)	5 (10%)	0.26
Heart failure	19 (20%)	14 (28%)	5 (10%)	0.04
History of stroke	5 (5%)	4 (8%)	1 (2%)	0.19
Vascular disease	4 (4%)	2 (2%)	2 (2%)	1.00
CHA_2_DS_2_‐VASc score	2 (1, 3)	2 (1, 3)	2 (1, 3)	0.16
Antiarrhythmic drug use	48 (49%)	28 (56%)	20 (42%)	0.18
Class I	29 (29%)	14 (28%)	15 (31%)	0.67
Class III	4 (4%)	2 (4%)	2 (4%)	1.00
Class IV	24 (24%)	16 (32%)	8 (17%)	0.08
Echocardiographic measurements				
LVEF (%)	64 ± 11	61 ± 13	66 ± 8	0.01
LAd (mm)	39 ± 6	40 ± 6	38 ± 6	0.03

*Note:* Values are shown as the mean ± SD, median (25th, 75th interquartile range) or *n* (%).

Abbreviations: AF, atrial fibrillation; BMI, body mass index; LAd, left atrial dimension; LVEF, left ventricular ejection fraction.

### 3.2 Effective and Safety Outcome

**FIGURE 3:**
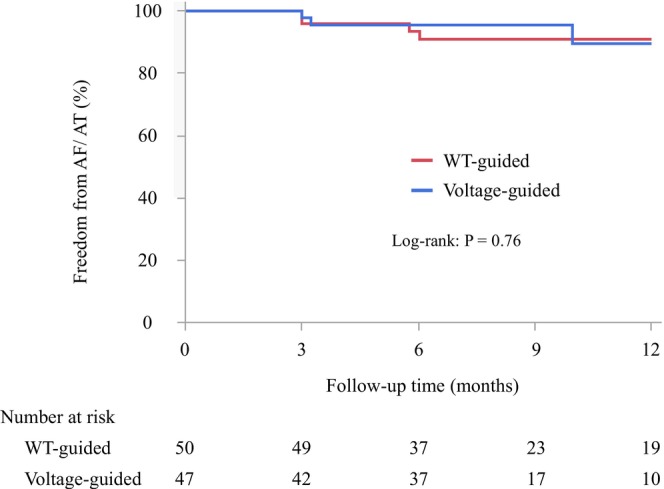
Kaplan–Meier curves for freedom from AF/AT recurrence. WT, Wall thickness.

All the patients achieved successful PVI. Procedure‐related variables and acute clinical outcomes are shown in Table 2. In all patients, the first‐pass PVI, APVR, and PV gaps (defined as the combined occurrence of first‐pass failure and/or APVR) were observed in 77 (79%), 16 (16%), and 28 (29%), respectively. The first‐pass PVI rate tended to be higher in the WT‐guided group compared to the Voltage‐guided group (43 [86%] vs. 34 [72%], *p* = 0.09), and the incidence of APVR tended to be lower (5 [10%] vs. 11 [23%], *p* = 0.07). Consequently, the proportion of patients with PV gaps was significantly lower in the WT‐guided group (10 [20%] vs. 18 [38%], *p* = 0.04). Because of greater LAd, the ablation line required for RPV isolation was significantly longer in the WT‐guided group than in the Voltage‐guided group. There were no significant differences in other variables such as total procedure time, PVI time, mapping time, fluoroscopic time, the ablation line required for LPV isolation, the number of RF application points, Extra‐PV ablation, and ablation procedure‐related complications between the WT‐guided group and the Voltage‐guided group (All *p* > 0.05). Ablation parameters were shown in Table 3. In lesions delivered at 90 W for 4 s, the WT‐guided group exhibited a significantly lower mean impedance drop (7.4 ± 2.7 vs. 7.9 ± 3.2, *p* < 0.01) and a significantly higher total RF energy delivery compared to the Voltage‐guided group (319 ± 36 J vs. 306 ± 72 J, *p* < 0.01). No significant differences were observed in the remaining ablation parameters between the two groups for the 90 W/4 s lesions. In contrast, for lesions delivered at 50 W, the mean contact force was significantly greater in the WT‐guided group than in the Voltage‐guided group (13 ± 5 g vs. 13 ± 5 g, *p* = 0.02), whereas no significant differences were noted in the other ablation parameters. During a median follow‐up period of 6.5 months (interquartile range, [6–12] months), the Kaplan–Meier analysis showed no significant difference in the 1‐year freedom from AF/AT between the groups (92% vs. 94%, log‐rank *p* = 0.76) (Figure 3).

**TABLE 2:** Procedure‐related variables and acute clinical outcomes.

Procedure‐related variables	Total (*n* = 97)	WT‐guided (*n* = 50)	Voltage‐guided (*n* = 47)	*p*
Procedure time (min)	109 ± 34	109 ± 33	110 ± 36	0.85
PVI procedure time (min)	35 ± 13	33 ± 10	36 ± 15	0.21
LA mapping time (min)	8 ± 7	8 ± 2	9 ± 3	0.10
Fluoroscopic time (min)	8 ± 7	8 ± 6	8 ± 8	0.96
Ablation line for LPV isolation (mm)	121 ± 16	122 ± 15	119 ± 17	0.33
Ablation line for RPV isolation (mm)	119 ± 15	122 ± 16	116 ± 14	0.03
Number of RF application points	74 ± 15	72 ± 15	75 ± 15	0.40
90 W/4 s	33 ± 17	35 ± 17	31 ± 18	0.25
50 W (target AI: 450–500)	40 ± 18	36 ± 15	44 ± 20	0.06
Extra‐PV ablation				
LA posterior isolation	10 (10%)	7 (14%)	3 (6%)	0.31
Mitral isthmus ablation	2 (2%)	0	2 (2%)	0.23
SVC isolation	7 (7%)	6 (12%)	1 (2%)	0.11
CTI ablation	22 (22%)	12 (24%)	10 (21%)	0.74
Acute clinical outcome				
First‐pass PVI rate	77 (79%)	43 (86%)	34 (72%)	0.09
Acute PV reconnection	16 (16%)	5 (10%)	11 (23%)	0.07
PV gaps	28 (28%)	10 (20%)	18 (38%)	0.04
Complication	0	0	0	1.00

*Note:* Values are shown as the mean ± SD, median (25th, 75th interquartile range) or *n* (%).

Abbreviations: AI, ablation index; LPV, left pulmonary vein; PV, pulmonary vein; PVI, pulmonary vein isolation; RF, radiofrequency; RPV, right pulmonary vein; SVC, superior vein isolation; WT, wall thickness.

**TABLE 3:** Ablation related parameters.

*n* = 1552 segments (97 patients)	WT‐guided (*n* = 784)	Voltage‐guided (*n* = 768)	*p*
90 W/4 s lesions			
Mean contact force (g)	17 ± 8	17 ± 8	0.64
Mean impedance drop (Ω)	7.4 ± 2.7	7.9 ± 3.2	< 0.01
FTI (gs)	66 ± 31	65 ± 30	0.17
Max temperature (°C)	51 ± 3	51 ± 3	< 0.01
Total RF energy (J)	319 ± 36	306 ± 72	< 0.01
50 W (target AI: 450–550) lesions			
Mean contact force (g)	13 ± 5	13 ± 5	0.02
Mean impedance drop (Ω)	7 ± 3	7 ± 3	0.83
FTI (gs)	178 ± 73	181 ± 75	0.19
AI	452 ± 73	456 ± 71	0.07
Max temperature (°C)	45 ± 2	45 ± 2	0.08

*Note:* Values are shown as the mean ± SD or *n* (%).

Abbreviations: AI, ablation index; FTI, force time integral; RF, radiofrequency; WT, wall thickness.

### 3.3 Relationship Between PV Gaps, Patient Characteristics, Ablation Strategies, RF Output Setting, and LAWT

**FIGURE 4:**
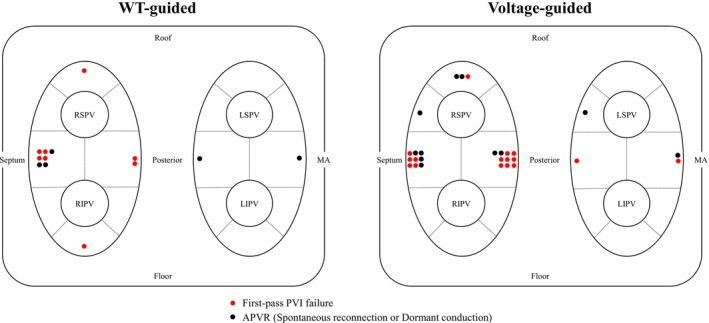
Distribution of pulmonary vein (PV) gaps. Red circles indicated first‐pass PV isolation failure sites, and black circles represent sites of acute PV reconnection. APVR, acute pulmonary vein re connection; LIPV, left inferior pulmonary vein; LSPV, left superior pulmonary vein; MA, mitral anulus; RIPV, right inferior pulmonary vein; RSPV, right superior pulmonary vein; WT, wall thickness.

The distribution of PV gaps in the 16 PV segments for each group is shown in Figure 4. PV gaps were observed in all PV segments in 40 of 1552 segments (2%) in 28 patients (28%). Patients with PV gaps were significantly younger (62 ± 12 vs. 67 ± 10, *p* = 0.03) and had a significantly lower CHA_2_DS_2_‐VASc score (1 [1, 2.75] vs. 2 [1, 3]) compared to those without PV gaps. There were no differences in other patient characteristics including LVEF and LAd between patients with and without PV gaps (Table S1). Among the 1552 segments analyzed, the number of PV gap segments was significantly lower in the WT‐guided group compared to the Voltage‐guided group (14 of 784 segments [1%] vs. 26 of 768 [3%], *p* = 0.04). No significant difference was observed between the two groups in the number of segments ablated with 90 W for 4 s (389 of 784 segments [49%] vs. 347 of 768 [47%], *p* = 0.08).

In the analysis of 1264 segments from 79 patients who underwent contrast‐enhanced CT, there was no significant difference in the proportion of segments with an LAWT ≥ 2 mm between the WT‐guided and Voltage‐guided groups (386 of 752 segments [51%] vs. 266 of 512 [51%], *p* = 0.82). Univariate and multivariate logistic regression analyses for PV gap segments are shown in Table 4. Univariate analysis identified the carina segments and LAWT ≥ 2 mm segments as significant clinical variables associated with PV gaps. In the multivariate analysis, both the carina segments (OR 15.40, 95% CI 6.06–39.30, *p* < 0.01) and the Voltage‐guided group (OR 2.10, 95% CI 1.01–4.37, *p* = 0.04) remained independently associated with PV gap formation.

**TABLE 4:** Univariate and multivariate logistic regression analysis for predictors of PV gap segments.

	Univariate analysis		Multivariate analysis	
	Odds ratio (95% CI)	*p*	Odds ratio (95% CI)	*p*
Voltage‐guided (vs. WT‐guided)	1.92 (0.99–3.71)	0.05	2.10 (1.01–4.37)	0.04
Carina segments (vs. non‐carina segments)	15.00 (6.73–35.00)	< 0.01	15.40 (6.06–39.30)	< 0.01
90 W/4 s (vs. 50 W)	0.52 (0.27–1.02)	0.06	2.07 (0.84–5.08)	0.11
LAWT > 2 mm (vs. ≤ 2 mm)	2.67 (1.23–5.77)	0.01	1.70 (0.62–4.63)	0.29

Abbreviations: LAWT, left atrial wall thickness; PV, pulmonary vein.

Among 40 PV gap segments, contrast‐enhanced CT data were available in 34 segments. Of these, 13 were ablated with 90 W/4 s and 21 with 50 W. When 90 W/4 s was applied, segments with LAWT ≥ 2 mm were significantly more frequent in the Voltage‐guided group than in the WT‐guided group (8/10 [80%] vs. 0/3, *p* = 0.035). These eight segments had a low mean bipolar voltage of 0.8 ± 0.5 mV. In contrast, for segments ablated with 50 W, the proportion of LAWT ≥ 2 mm did not differ between Voltage‐guided and WT‐guided groups (8/10 [80%] vs. 9/11 [81%]). In segments with LAWT < 2 mm, conduction gaps were observed in 9/34 (26%) across both groups (4 in the Voltage‐guided group vs. 5 in the WT‐guided group). These sites were mainly located at the anterior carina of the right PVs (*n* = 5), followed by the right PV roof (*n* = 2), the posterior carina (*n* = 1), and the inferior aspect of the right PVs (*n* = 1).

## 4 Discussion

### 4.1 Main Findings

This study had three significant findings. First, the first‐pass PVI rate tended to be greater in the WT‐guided group than the Voltage‐guided group, and the PV gaps (defined as the combined occurrence of first‐pass failure and/or APVR) were significantly greater in the WT‐guided group than in the Voltage‐guided group. Second, multivariable‐adjusted analysis demonstrated that WT‐guided ablation was significantly more effective than Voltage‐guided ablation in preventing PV gaps. Third, the 1‐year recurrence rates of AF/AT were comparable between the WT‐guided and Voltage‐guided ablation groups.

### 4.2 Acute PV Gaps Between WT‐Guided Group vs. Voltage‐Guided Group

The PV gaps were significantly lower in the WT‐guided group compared to the Voltage‐guided group. Several potential mechanisms may account for this finding. First, lesion formation during RF ablation is highly dependent on the power and duration of energy delivery. Preclinical studies have demonstrated that lesion depth increases in the order of low‐power, long‐duration, HPSD, and vHPSD ablation [2, 7]. Conversely, lesion width increases in the reverse order, with the broadest lesions generated by very high‐power, short‐duration ablation [2, 7]. Our prior retrospective observational study further identified LAWT ≥ 2.3 mm as the strongest predictor of PV gap formation in vHPSD PVI. These findings suggest that achieving durable transmural lesions with vHPSD may be challenging in regions of increased wall thickness. The WT‐guided ablation protocol used in the present study likely optimized lesion formation by tailoring energy application to the LAWT, thereby enhancing procedural efficacy while mitigating excessive ablation. Second, the observed difference may also be attributed to differences in the ablation protocols. In our previous study investigating predictors of PV gap formation during vHPSD PVI, a bipolar voltage > 2.4 mV and a LAWT > 2.3 mm were identified as independent predictors [6]. In the current study, the WT‐guided protocol applied 50 W energy delivery to regions with wall thickness > 2.0 mm, ensuring sufficient lesion depth in thicker tissue. In contrast, the Voltage‐guided protocol applied 50 W ablation only to sites with bipolar voltage > 2.4 mV, which resulted in the use of the default 90 W/4 s setting for areas with modestly low voltage—even when those areas had increased wall thickness, such as 2.2 or 2.1 mm. As a result, regions that may have required 50 W ablation to achieve durable lesion formation were instead treated with a higher power. However, this phenomenon is not merely a consequence of protocol design; rather, it reflects the inherent discordance between LAWT and bipolar voltage. Indeed, our previous investigation reported only a modest and inconsistent correlation between these two parameters [6, 8, 9, 10]. In the Voltage‐guided group of the present study, ablation at 8 sites characterized by low bipolar voltage (mean voltage: 0.8 ± 0.5 mV) but increased LAWT > 2 mm using a 90 W/4 s setting resulted in a high incidence of conduction gaps. The previous study has reported that in the setting of progressive atrial remodeling, bipolar voltage tends to decrease while wall thickness increases at corresponding sites [11]. Furthermore, Shinzato et al. [12] demonstrated that patients with AF exhibiting extensive atrial amyloid deposition on biopsy were more likely to have low‐voltage areas in the LA voltage maps. These findings imply that in patients with advanced atrial remodeling, including those with amyloid infiltration, low‐voltage regions may paradoxically exhibit increased wall thickness, potentially impairing the thermal conductivity of RF energy and reducing ablation efficacy. In contrast, only 9 (26%) gap segments were observed in thinner regions (LAWT < 2 mm), where multiple procedural or anatomical factors—such as insufficient contact, carina or roof anatomy, epicardial fat, or epicardial connections—likely contributed. These findings indicate that while various mechanisms can lead to lesion failure, their relative contribution is limited compared with the dominant effect of increased wall thickness.

Conversely, in the present study, twenty‐one segments occurred despite ablation with 50 W. In both the WT‐guided and Voltage‐guided groups, PV gaps were frequently observed in the carina region. This may reflect the anatomic complexity of the PV carina and the involvement of epicardial conduction, which has been reported to be identifiable by activation mapping during sinus rhythm [13]. Verifying the presence of early‐activated epicardial conduction before PVI and tailoring the ablation carina line to eliminate such pathways may further improve procedural efficacy. Moreover, given the frequent contribution of epicardial structures at the carina, low‐power, long‐duration applications should also be considered in this region.

### 4.3 Clinical Implications

In this study, both WT‐guided and voltage‐guided ablation achieved favorable first‐pass PVI rates (86% vs. 72%) compared to previous vHPSD outcomes [3, 4]. WT‐guided ablation, using ADAS software for automated wall thickness assessment, may offer superior acute lesion durability, especially in patients with advanced LA remodeling where voltage and wall thickness are not well correlated [11]. Moreover, since pre‐procedural CT enables direct lesion planning, additional intra‐procedural mapping can be minimized, potentially shortening overall procedure time.

However, WT‐guided ablation requires preprocedural CT and may be less feasible in patients with asthma, renal dysfunction, or without access to ADAS. Voltage‐guided ablation is widely feasible, relying on standard mapping systems [14], but may be less effective in thick‐walled, low‐voltage regions, potentially leading to shallow lesions [11]. Accurate voltage mapping also requires stable SR. Despite these differences, both strategies showed similarly low AF/AT recurrence at 1 year, supporting the efficacy of either approach.

### 4.4 Limitations

This study has several limitations. First, it was a small, prospective observational study, and the choice of ablation strategy—WT‐guided or voltage‐guided—was non‐randomly assigned, which may have introduced selection bias. Although some degree of bias may exist, patient characteristics were not associated with PV gap formation. However, baseline characteristics such as age and CHA_2_DS_2_‐VASc score did not significantly differ between the two groups. Minor differences were observed in LAd and LVEF, but these were not associated with PV gap formation in the multivariate analysis. While some differences in ablation parameters, such as impedance drop and maximum temperature, were observed between the two groups (Table 3), these differences likely reflect inherent differences in the sites selected for ablation based on the respective guiding strategies (Voltage vs. Wall Thickness), rather than differences in ablation quality or operator technique. After adjusting for significant procedural factors in the multivariate analysis, Voltage‐guided ablation remained independently associated with PV gap occurrence. Second, in our previous report, predictors of PV gaps with vHPSD ablation were LAWT > 2.3 mm and bipolar voltage > 2.4 mV [6]. In the WT‐guided group of the present study, we applied HPSD to regions with LAWT > 2.0 mm, since ADAS 3D CT displays LA wall thickness in 1‐mm increments. In contrast, the Voltage‐guided group could apply HPSD to sites with bipolar voltage > 2.4 mV. This difference in thresholds for HPSD applications may have contributed to the observed disparity in PV gaps. If the threshold in the Voltage‐guided group had been set at 2.0 mV, the gap incidence might have been reduced. Third, patients who could not undergo contrast‐enhanced CT were assigned only to the voltage‐guided group. In addition, voltage maps could not be obtained in some patients due to IRAF. These factors may have affected group comparability. A randomized controlled trial is needed to address these biases and more rigorously compare the two strategies. Finally, the generalizability of our findings remains limited. Wall thickness–guided ablation is not yet a widely adopted standard, and the use of 90 W for 4 s remains debated. Furthermore, although the sample size is respectable for a single‐center study, it restricts the statistical power to detect differences in secondary outcomes such as 1‐year AF/AT recurrence. Therefore, our findings should be considered hypothesis‐generating and require confirmation in larger randomized studies.

## 5 Conclusions

WT‐guided ablation using integrated pre‐procedural CT imaging and ADAS 3D software was associated with a significantly lower incidence of PV gaps than a conventional bipolar voltage‐guided strategy despite similar 1‐year clinical outcomes. These findings support individualized, LA wall thickness–based strategies in the high‐power RF ablation era.

## Ethics Statement

The studies involving human participants were performed according to protocols approved by the Institutional Review Board of Nihon University Itabashi Hospital (RK‐230314‐14).

## Consent

Informed consent was obtained through an opt‐out process, in accordance with institutional and ethical guidelines.

## Conflicts of Interest

Dr. Watanabe has received research funding from Fukuda Foundation for Medical Technology. Dr. O.Y. has received research funding from Biosense Webster Inc., scholarship donation from Boston Scientific Japan, and Endowed Courses from Boston Scientific Japan, Japan Lifeline, Fukuda Denshi, Abbott Japan, BIOTRONIK Japan, Medtronic Japan. K.N. has received research funding and accepted remuneration from Johnson & Johnson. The other authors declare no conflicts of interest.

## Supporting information


**Table S1:** Patient characteristics with PV gaps and without PV gaps.

## Data Availability

The data that support the findings of this study are available on request from the corresponding author. The data are not publicly available due to privacy or ethical restrictions.
