# Supplementary material for: Wall Thickness‐Guided vs. Voltage‐Guided Pulmonary Vein Isolation for Atrial Fibrillation
Source: J Arrhythm. 2025 Oct 27;41(5):e70215. doi: 10.1002/joa3.70215 (PMC12559026; doi:10.1002/joa3.70215)
Supplement: Supplementary file 1 — Table S1: Patient characteristics with PV gaps and without PV gaps. [file JOA3-41-e70215-s001.docx]

| **Supplemental table. Patient characteristics with PV gaps and without PV gaps** | | | |
| --- | --- | --- | --- |
|  | Patients  with PV gaps  (n=28) | Patients  without PV gaps  (n=69) | P value |
| **Clinical characteristics** |  |  |  |
| Age (years) | 62 ± 12 | 67 ± 10 | 0.03 |
| Male sex | 21 (75%) | 42 (60%) | 0.18 |
| BMI (kg/m^2^) | 24 ± 3 | 23 ± 4 | 0.43 |
| Paroxysmal AF | 18 (64%) | 43 (62%) | 0.85 |
| AF duration (months) | 19 ± 27 | 20 ± 41 | 0.93 |
| Hypertension | 12 (42%) | 39 (56%) | 0.77 |
| Hyperlipidemia | 4 (14%) | 9 (13%) | 0.87 |
| Diabetes mellitus | 3 (10%) | 12 (17%) | 0.39 |
| Heart failure | 5 (17%) | 14 (20%) | 0.78 |
| History of stroke | 3 (10%) | 3 (2%) | 0.14 |
| Vascular disease | 1 (3%) | 3 (4%) | 1.00 |
| CHA_2_DS_2_-VASc score | 1 (1, 2.75) | 2 (1, 3) | 0.03 |
| Antiarrhythmic drug use | 16 (57%) | 32 (46%) | 0.33 |
| Class I | 11 (39%) | 18 (26%) | 0.19 |
| Class III | 2 (7%) | 2 (2%) | 1.00 |
| Class IV | 7 (17%) | 17 (24%) | 1.00 |
| **Echocardiographic measurements** |  |  |  |
| LVEF (%) | 64 ± 10 | 64 ± 12 | 0.92 |
| LAd (mm) | 38 ± 5 | 39 ± 5 | 0.42 |
| Values are shown as the mean ± SD, median (25th, 75th interquartile range) or n (%).  AF, atrial fibrillation; BMI, body mass index; LAd, left atrial dimension; LVEF, left ventricular ejection fraction. | | | |
